# Use of Saponinosomes from *Ziziphus spina-christi* as Anticancer Drug Carriers

**DOI:** 10.1021/acsomega.2c03109

**Published:** 2022-08-04

**Authors:** Zahra Nazemoroaya, Mohsen Sarafbidabad, Athar Mahdieh, Darya Zeini, Bo Nyström

**Affiliations:** † Student Research Committee, School of Pharmacy, Shahid Beheshti University of Medical Sciences, 19839-63113 Tehran, Iran; ‡ Department of Biomedical Engineering, Faculty of Engineering, University of Isfahan, 81746-73441 Isfahan, Iran; § School of Pharmacy, Department of Pharmaceutics, University of Oslo, P.O. Box 1068, Blindern, N-0316 Oslo, Norway; ∥ Department of Chemistry, University of Oslo, P.O. Box 1033, Blindern, N-0315 Oslo, Norway; ⊥ Laboratory of Neural Development and Optical Recording (NDEVOR), Department of Molecular Medicine, Institute of Basic Medical Sciences, University of Oslo, P.O. Box 1103, N-0317 Oslo, Norway

## Abstract

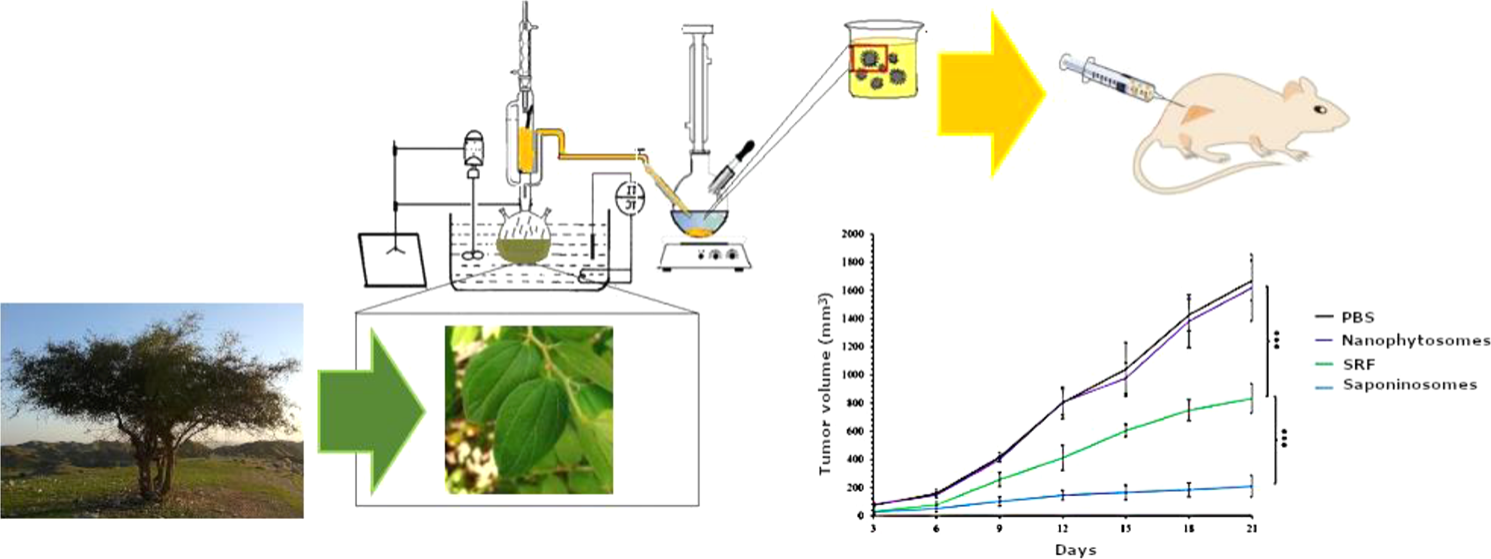

Saponins are plant glycosides with different structures and biological activities, such as anticancer effects. *Ziziphus spina-christi* is a plant rich in saponin, and this compound is used to treat malignant melanoma in the present study. Nanophytosomes can be used as an advantageous nanodrug delivery system for plant extracts. The aim of this work is to use the saponin-rich fraction (SRF) from *Z. spina-christi* and prepare SRF-loaded nanophytosomes (saponinosomes) and observe the in vitro and in vivo effects of these carriers. First, the SRF was obtained from *Z. spina-christi* by a solvent–solvent fractionation method. Then, Fourier transform infrared (FTIR) analyses were performed to confirm the presence of saponins in the extracted material. Subsequently, the saponinosomes were prepared by the solvent injection method (ether injection method) using a 1:1:1 ratio of lecithin/cholesterol/SRF in the mixture. Characterization of the prepared saponinosomes was performed by FTIR, dynamic light scattering (DLS), field-emission scanning electron microscopy (FE-SEM), and atomic force microscopy (AFM) analyses. In addition, a UV–vis spectrophotometer was used to determine the entrapment efficiency (EE) and in vitro release of the SRF. Finally, cell cytotoxicity of the different formulations was evaluated using a 3-[4,5-dimethylthiazol-2-yl]-2,5-diphenyl tetrazolium bromide (MTT) assay on both mouse melanoma cells (B16F10) and fibroblasts (L929). Using DLS, AFM, and FE-SEM analyses, the particle size was determined to be 58 ± 6 nm with a zeta potential of −32 ± 2 mV. The calculated EE was 85 ± 3%. The results of the in vitro release profile showed that 68.2% of the SRF was released from the saponinosome after 48 h. The results of the MTT assay showed that the SRF and saponinosomes have high toxicity on B16F10 melanoma cells, but saponinosomes showed a significant decrease in cytotoxicity on L929 fibroblast cells compared with that of the SRF. Our results indicate that the SRF from *Z. spina-christi* has anticancer activity, and the saponinosomes prepared in this work can control tumor growth, improve therapeutic efficacy, and reduce the side effects of saponins.

## Introduction

Cancer is a significant universal health issue and one of the leading death causes in the world.^1^ Natural resources such as plants have long been considered as a source of chemotherapeutic or chemopreventive agents against cancer.^2−4^ However, due to the increasing resistance of certain tumors and the severe side effects of conventional chemotherapy, new pharmacological molecules are needed. In recent decades, advances in cancer molecular biology have identified several biological compounds capable of inhibiting cancer cell growth with improved efficacy and selectivity.^5−11^ Structural diversity and associated synergistic effects, high efficiency, availability, and excellent biocompatibility are the advantages of plants as medicinal resources. Traditional plants contain phytochemical compounds, which are mainly secondary metabolites used by plants to ensure their survival and fertility. Phytochemical compounds of medicinal importance include glucosinolates, alkaloids, triterpenoids, flavonoids, saponins, pigments, and tannins. The use of plant secondary metabolites in traditional medicine has been reported in many studies. These secondary metabolites showed various biological activities, such as antimicrobial,^12−14^ anti-inflammatory,^12^ antiviral,^13^ and anticancer properties.^13^

Saponins are one of the most important groups of secondary metabolites widely distributed in various plant species. Saponins consist of two parts, aglycone as the nonsugar part, which is hydrophobic, and one or more sugar chains associated with the aglycone part. The variation in the structure and properties of saponins depends on their aglycone type, degree of hydroxylation, and type and number of sugar chains.^15,16^ Several studies have been conducted to characterize the properties of saponins, such as antitumor, antibacterial, antiviral, antifungal, antidiabetic, antioxidant, and anti-inflammatory effects.^17−19^ The profound effects of saponins on cancer cells have attracted considerable attention in the medical and pharmaceutical fields. Saponins from plants have shown a high potential to inhibit various cancer cells under in vitro and in vivo conditions.^20−22^ Despite the significant progress made in recent years, the use of saponins as anticancer agents has certain drawbacks, mainly due to their cytotoxicity, poor pharmacokinetic properties, low bioavailability, and low penetration across the cell membrane.^3^

Nowadays, much attention has been paid to the antitumor effect of saponins, and several publications have appeared.^7−11,23^ Some possible reasons for the antitumor effect of saponins are the formation of pores in cell membranes and thus increased permeabilization, induction of apoptosis, inhibition of angiogenesis and metastasis, and reduction of drug efflux.^24^ Although many studies have shown that saponins damage tumors rather than attack normal organs, their use as antitumor agents in clinical trials is a major obstacle because of their high organ toxicity.^25^ The hemolytic activities of saponins are mediated by permeabilization of the erythrocyte membrane via an interaction with plasma membrane cholesterol.^26^ This activity is associated with critical carboxyl and hydroxyl groups of the triterpenoid saponins. Because of their amphiphilic properties, saponins have the potential to form pores in biological membranes and alter cell permeabilization,^27^ and they can be considered as hemolytic agents. This is due to the formation of a saponin–cholesterol complex in the cell membrane. The activity of saponins is dose-dependent, and a significant increase in dose would lead to a marked increase in bioavailability and effect, which may significantly increase the cytotoxicity of saponin.^25,26^

Certain limitations of herbal drugs and phytochemicals, such as instability at low acidic pH, presystemic metabolism in the liver, solubility, and absorption problems, may cause the drug concentration in plasma to be below the therapeutic concentration, resulting in a lower therapeutic effect. However, the use of novel drug delivery technologies for herbal drug sources reduces the presystemic metabolism, degradation of the drug in the gastrointestinal tract, and distribution/accumulation of the drug in the nontargeted tissues and organs. This approach reduces side effects and improves therapeutic efficacy and ultimately patient compliance.^28^ Several novel drug delivery systems have been used for herbal drugs and phytochemicals.^29^ Typical carriers for phytochemicals can be classified as follows: vesicular delivery systems (liposomes, ethosomes, phytosomes, and transferosomes^30^), particulate delivery systems (microspheres, nanoparticles, and micropellets), and biphasic systems (micro-/nanoemulsions).^31−33^ With this in mind, many researchers have focused on developing more efficient delivery systems for the specific delivery of saponins and their release at the tumor site. To this end, various approaches in the preparation of carriers, such as the synthesis of solid lipid nanoparticles, liposomes, phytosomes, and nanoparticulate saponin bases, have been investigated.^34−38^ Among these methods, phospholipid conjugation with a saponin extract has many advantages and superiorities over the others. For example, phospholipids are biocompatible, safe, and hepatoprotective components that can improve targeting, stability, bioavailability, biocompatibility, and therapeutic efficacy.^39,40^

Targeted drug delivery is an alternative approach that should be further explored to increase the efficacy of saponins. Nanoparticles may evade clearance by plasma-binding proteins and the reticuloendothelial system due to their size.^41^ Nanoencapsulation not only prolongs the drug’s circulation time but also reduces cytotoxicity to normal cells. For instance, in one project, herbal drugs and incorporation of saponins into nanocomposites of human serum albumin resulted in improved anticancer drug efficacy and no cytotoxicity to normal cells.^42^ In another project, saponin-loaded chitosan nanoparticles (nanosaponin) showed specific toxicity to cancer cells, whereas they were nontoxic to normal cells.^37^

The lipid bilayer membrane is normally composed of phospholipids. Phospholipids are biocompatible, nontoxic, and hepatoprotective.^39^ Hydrophilic phytoconstituents can be complexed with clinically useful nutrients such as phospholipids to convert them into lipid-soluble complexes. These complexes can be utilized to produce liposome-like vesicles called phytosomes. In phytosomes, the complexation of phospholipids and water-soluble active plant constituents is accompanied by the formation of hydrogen bonds, which is why they are more stable, whereas in liposomes, no chemical bond is formed. Phytosomes significantly improve the bioavailability of these hydrophilic active components. Phytosomes can easily cross lipid membranes and are reported to increase the bioavailability of poorly lipid-soluble herbal drugs by enhancing absorption in the gastrointestinal tract. The complexes of plant constituents and phospholipids are known as phytosomes, which are structurally similar to liposomes but smaller in size.^43^ Phospholipids can react with OH groups in plant extract constituents; this may lead to increased bioavailability, stability, and reduction in the cytotoxicity of plant extracts such as saponin extracts.^44^

The leaves of *Ziziphus spina-christi* have been known since ancient times as a medicinal plant and soap for skin treatment. Four triterpenoid saponin glycosides have been identified from the *n*-butanol extract of *Z. spina-christi* leaves, designated as christinin-A, christinin-B, christinin-C, and christinin-D.^45^ Because the use of phospholipids can improve bioavailability and increase the absorption of phytoconstituents, loading saponin extracts into nanophytosome carriers may improve efficacy in various medicinal applications.

In this study, saponin-rich fractions (SRFs) were prepared from *Z. spina-christi,* and the corresponding SRF-loaded nanophytosomes (saponinosomes) were synthesized by the ether injection method.^46^ One aim of the present work is to prepare saponinosome carriers and to investigate the effect of cytotoxicity of the SRF and the loaded carriers. In addition, the effects of the SRF on the murine melanoma cell line (B16F10) and fibroblast cell line (L929) were investigated. Finally, in vivo experiments were performed on mice infected with malignant melanoma, and it was observed that tumor growth was significantly inhibited by the addition of saponin-loaded carriers. The results of this research introduced a useful natural product that can be used as a nanodrug delivery system for cancer treatment.

## Experimental Section

### Materials

Soybean lecithin type IV-S (l-α-phosphatidylcholine, 1, 2-diacyl-*sn*-glycero-3-phosphocholine, 3-*sn*-phosphatidylcholine, l-α-lecithin, and azolectin) and cholesterol grade > 99% (3β-hydroxy-5-cholestene) were all purchased from Sigma-Aldrich Co. (St. Louis, MO, USA). All solvents (analytical grade) were obtained from Merck (Germany).

### Collection and Treatment of the Plant

The dried leaves of *Z. spina-christi* were obtained from tropical areas in the south of Iran. The collected leaves were washed several times (until no visible color of the water was detected) with distilled water to remove impurities and dried at room temperature for 10 days. The dried leaves were then pulverized to a fine powder using a laboratory mill, sieved with a mesh size of 2 mm, and stored in a dry, airtight container for further use.

### Extraction and Preparation of the SRF

**Figure 1:**
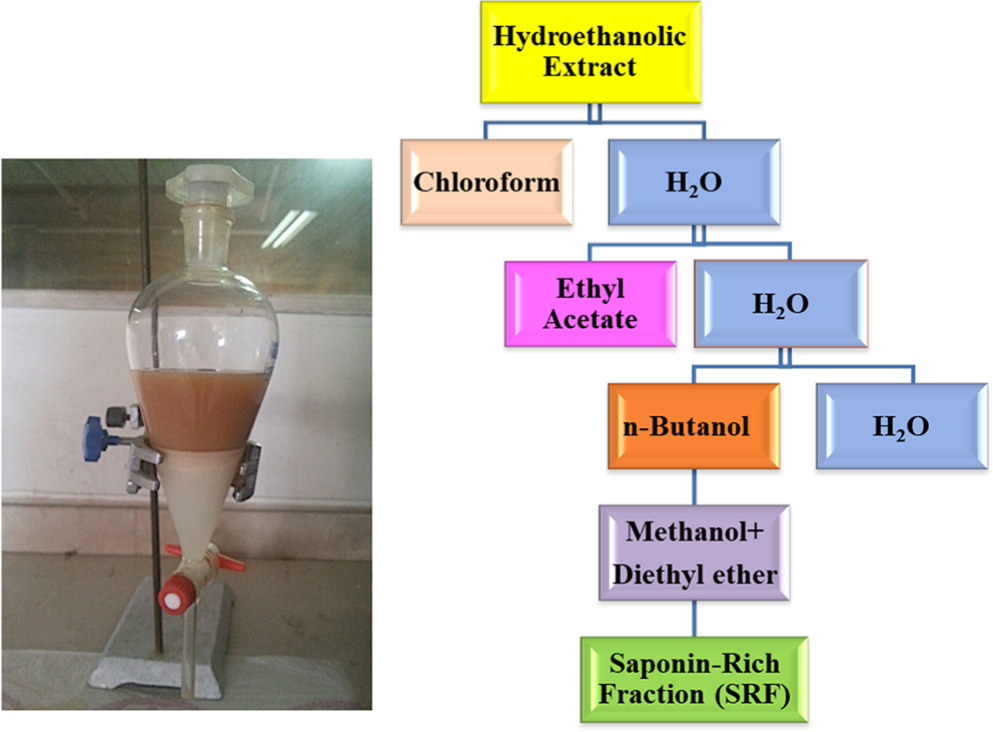
Schematic illustration of the solvent–solvent fractionation of the hydroethanolic extract of *Z. spina-christi* leaves.

The leaf material was extracted using ethanol as the extractant in the ratio of 1:1 of powdered leaves and ethanol using a Soxhlet extractor at 65 °C. The supernatant was removed from the residue by filtration using a Whatman no. 1 filter paper. This procedure was repeated five times to extract the plant material completely. The solvent was evaporated under vacuum using a rotary vacuum evaporator at 65 °C for 45 min. Solvent–solvent fractionation of the hydroethanolic extract was carried out using different solvents. First, the hydroethanolic extract was partitioned between chloroform–water mixtures in a separating funnel with equal proportions. After 30 min, ethyl acetate (EtOAc) was added to the aqueous phase with vigorous shaking. The EtOAc fraction was separated after 30 min, and the aqueous phase was saturated with *n*-butanol. In the next step, the butanolic fraction was dehydrated with anhydrous sodium sulfate and concentrated under reduced pressure, and the residue was dissolved in methanol. The SRF was obtained by precipitation with the addition of anhydrous diethyl ether, and the precipitates were dried and used for further experiments. All extraction steps were repeated five times at room temperature with equal proportions of solvents. The fractionation protocol is shown in Figure 1.

### Synthesis of Saponinosomes

The SRF was used to prepare saponinosomes. These species were synthesized at a mass ratio of 1:1:1 of SRF/cholesterol/lecithin by using the ether injection method.^45^ This technique involves the interaction of lipids dissolved in an organic solvent with herbal extracts in the aqueous phase. In the first step, the SRF was dissolved in phosphate-buffered saline (PBS) buffer as an aqueous phase in a round-bottom flask at a temperature of 65 °C. Then, soybean lecithin and cholesterol were dissolved in diethyl ether as an organic phase and slowly injected dropwise into the aqueous phase in the round-bottom flask. Then, stirring was continued at this temperature until the organic solvent had evaporated. After cooling the solution to room temperature, the solution was centrifuged at 20,000 rpm for 15 min to obtain the saponinosomes in this work. The nanophytosomes without the SRF (blank nanophytosomes) were prepared by the same procedure but in the absence of the SRF.

### Characterization Methods

Characterization of the prepared samples was performed by Fourier transform infrared (FTIR) spectroscopy (Jasco-6300, Japan), dynamic light scattering (DLS) (Malvern Zeta sizer), field-emission scanning electron microscopy (FE-SEM) (Mira Tescan), and atomic force microscopy (AFM) (DME Dualscop C-26).

### Entrapment Efficiency

The amount of the SRF loaded into the saponinosomes was estimated by a colorimetric method.^47,48^ 10 mg of the SRF was dissolved in 5 mL of distilled water, and 50 μL of the solution was distributed into different test tubes to which 0.25 mL of the vanillin reagent (8%, w/v in 99.9% ethanol) was added. The test tubes were placed in an ice-cold water bath, and 2.5 mL of 72% (v/v) sulfuric acid was slowly added to the reaction mixture. After the components were mixed in each test tube, the test tubes were allowed to stand for 3 min and then heated to 60 °C in a water bath for 10 min and then cooled in an ice-cold water bath. The colorimetric method for the SFR was used to obtain a standard reference curve. The same procedure used for the SRF was employed for the saponinosomes; they were put in the test tubes, and the absorbance was measured. The amounts of the SRF in the saponinosomes and the SRF content in the supernatant after centrifugation were obtained from the standard reference curve; finally, the entrapment efficacy was calculated (eq 1). The centrifugation of the samples was carried out at a speed of 20,000 rpm for 15 min. Measurements of the absorbance of the samples were made at 544 nm using a UV–vis spectrometer (Shimadzu V-570 Japan), and measurements of the absorbance of known concentrations of the compound at the same wavelength resulted in the establishment of a standard reference curve. The entrapment efficiency (EE, %) was calculated using the following equation
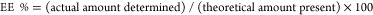
1

The actual amount of the SRF in the saponinosomes was calculated by subtracting the total SRF content used from the SRF content in the supernatant after centrifugation.

### In Vitro Release Profile

The release of the SRF from the saponinosomes was carried out by using the dialysis bag method, and PBS with a pH of 7.4 (physiological pH) was used as the release medium. The material of the dialysis tube was cellulose acetate. Briefly, a certain amount (1 mg) of the prepared saponinosome was dispersed in 1 mL of PBS, and the solution was transferred into the dialysis bag with a molecular weight cut-off of 3.5 kDa. The final concentration of saponinosomes in PBS was 1 mg/mL. The dialysis bag was immersed in 10 mL of PBS (pH 7.4). The dialysis bags were kept in an incubator shaker at 37 °C with a shaking frequency of 110 rpm. At predetermined intervals, 1 mL of the release medium was taken out and replaced with the same volume of fresh PBS. The amount of the SRF released from the carriers was determined by the aforementioned colorimetric method using a UV–vis spectrophotometer at a wavelength of 544 nm. The percentage of the SRF released was determined from the following equation using the prepared standard calibration curve of the SRF in PBS
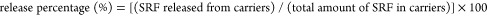
2

### Cell Culture

B16F10 (mouse melanoma) and L929 (mouse fibroblast) cell lines were purchased from the Pasteur Institute, Tehran, Iran. The B16F10 cancer cells and L929 normal cells were cultivated in 96-well microplates at a density of 10^4^ cells/well using Dulbecco’s modified Eagle’s medium (DMEM). The culture media were supplemented with fetal bovine serum at a final concentration of 10% and penicillin and streptomycin (a final concentration of 1%) in a humidified atmosphere of 5% CO_2_ and 95% air at 37 °C.

### Cell Viability Assay

The cytotoxic effect of the formulations against B16F10 and L929 cell lines was determined by a rapid colorimetric assay using 3-[4,5-dimethylthiazol-2-yl]-2,5-diphenyl tetrazolium bromide (MTT). In this assay, soluble MTT is converted to a water-insoluble colored formazan product by the mitochondrial enzyme activity of viable cells. The formazan is then dissolved in dimethyl sulfoxide (DMSO) and measured spectrophotometrically at a wavelength of 570 nm. Briefly, B16F10 and L929 cells were seeded in 96-well microplates at a density of 10^4^ cells/well, using complete cell culture media (DMEM), and incubated for 24 h. Then, the medium was replaced with 100 μL of the complete culture medium containing different concentrations of 5, 10, 20, 40, and 80 μg/mL (based on the SRF concentration) of the SRF, blank nanophytosomes (without the SRF), and saponinosomes incubated for 24 h under the same conditions. Since we wanted to compare the effects of the free SRF and SRF-loaded saponinosomes, the concentration of the SRF inside the saponinosomes was the same (5, 10, 20, 40, and 80 μg/mL) as that for the free SRF. To accomplish this situation, the EE of the SRF in saponinosomes was determined to be approximately 85%; from this value, the amount of the SRF loaded inside the saponinosomes could be calculated. For blank nanophytosomes, an equal amount of saponinosomes was utilized. For the control, wells containing only the cells in the medium without formulation were used. To evaluate cell survival, the medium was replaced with 50 μL of MTT solution (1 mg/mL in PBS) and incubated for 4 h. After that, 150 μM DMSO was added to each well to dissolve formazan precipitates and completely dissolve all formazan crystals formed. Absorbance measurements were then performed at a wavelength of 570 nm, while for the reference well, measurements were performed at a wavelength of 620 nm using the enzyme-linked immunosorbent assay plate reader. Cell viability was determined by comparing the absorbance of treated cells at each concentration with that of the corresponding control group. All measurements were carried out in triplicate.

### Fluorescence Microscopy

Qualitative cellular uptake of nanophytosomes was studied by fluorescence microscopy. Fluorescein-loaded nanophytosomes (F-nanophytosomes) were prepared by adding the fluorescein dye [FLUOCYNE 10% (sodium fluorescein 100 mg/mL)] as the tracking agent into the aqueous phase. The concentration of the dye in the formulation was 1 mg/mL, and the concentration of the formulation employed in the imaging experiments was 100 μg/mL. The fluorescein-containing carriers were prepared by the ether injection method as described above. In this case, 5 × 10^5^ B16F10 cancer cells were seeded in 6-well plates and incubated for 24 h. Cells were treated with a complete medium containing F-nanophytosomes. After different times (30 min, 6 h, and 12 h), the cells were washed three times with PBS and observed using a fluorescence microscope (IX71, Olympus, Japan).

### Flow Cytometry Analysis

Quantitative cellular uptake of nanophytosomes was assessed by flow cytometric analysis. B16F10 cancer cells were seeded in 6-well plates (5 × 10^3^ cells/well) and incubated for 24 h. Initially, cells were treated with a complete medium containing F-nanophytosomes. After 6 h incubation, cells were washed three times with PBS and detached with trypsin. The detached cells were centrifuged (1200 rpm, 5 min) and finally resuspended in 500 μL of PBS and analyzed using a flow cytometer (BD FACS Calibur, USA). The cells without any treatment were selected as a control group.

### In Vivo Animal Model and Treatment

For the in vivo experiments, 6–8 week old female C57BL/6 mice were purchased from the Iran Pasteur Institute. Animal experiments were performed in accordance with experimental guidelines approved by the Institutional Animal Care and Ethics committee. During the animal experiments, the animals were handled and cared for in a humane manner so that no additional pain or injury was inflicted on them. To minimize animal mortality during the experiments, only a limited number of animals were used to obtain statistically significant results.

Tumor models were generated by a subcutaneous injection of 2 × 10^6^ cells suspended in 50 μL of DMEM-F12 into the left flank of mice. The mice were used for treatment when the tumor volume reached 50 mm^3^. For the treatment, 150 μL of different formulations of PBS, SRFs, nanophytosomes, and saponinosomes was injected intraperitoneally into the mice every other day for 21 days. The injected doses were normalized to 15 mg/kg SRF. The tumor size was measured every other day using a digital caliper, and the tumor volume was calculated using the following equation^49^
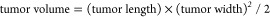
3

### Blood Chemistry and Histopathological Examination

After 21 days of treatment, one mouse from each group was sacrificed, and its blood was collected for serum chemical analysis. Plasma was discarded, and the concentrations of the components blood urea nitrogen (BUN), creatinine (Cr), alanine aminotransferase (ALT), aspartate aminotransferase (AST), and lactate dehydrogenase (LDH) were determined.

At the same time, the livers of the sacrificed mice were collected, fixed with 10% formalin, embedded in paraffin, and sliced into 5 μm sections. After staining the tissue sections with hematoxylin and eosin, their histopathology was assessed using a light microscope (BX51, Olympus, Japan) equipped with a digital camera (DP72, Olympus, Japan). In addition, Masson’s trichrome staining was used to detect possible liver fibrosis.

### Statistical Analysis

The Statistical Package for the Social Sciences (SPSS) software was used to plot the replicate experiments, and the results are presented as the mean value with standard deviation (mean ± SD). One-way analysis of variance (ANOVA) was used for statistical analysis; a value of *P* < 0.05 was considered significant (*n* = 3).

## Results and Discussion

### Extraction, Preparation, and Characterization of the SRF

The SRF was obtained from the leaf extract of *Z. spina-christi* by fractionation with different solvents, from nonpolar to polar solvents (Figure 1). Previous studies have shown that the butanolic fraction of *Z. spina-christi* is rich in saponins.^50^

The SRF samples studied in this work were extracted from the leaves of *Z. spina-christi* collected from the south of Iran. SRF samples from the leaves collected from the same area in Iran as well as saponin profiles from the leaf samples collected from different geographical areas were carefully analyzed recently.^45^ The structural characterization of the various saponins was performed using NMR, mass spectrometry (MS), and gas chromatography (GC)–MS. The results showed a complex composition of the studied *Z. spina-christi* leaves; 10 dammarane-type saponins and 12 phenolic compounds were identified. The analysis of all the samples showed that lotogenin glycosides were the main component in all studied samples, whereas konarigenin glycoside was present only in the leaf samples from the south of Iran.^45^

### Synthesis and Characterization of Saponinosomes

SRF–phospholipid complexes are known as precursors for the preparation of saponinosomes. Among the common methods for the synthesis of nanophytosomes based on plant extracts, the solvent injection method^51^ could be an ideal approach due to its simplicity, repeatability, and high EE of the plant extract. In this method, ether and ethanol injection techniques were used for different plant extracts. In this project, we used the ether injection technique, in which diethyl ether was used as the basic solvent for dissolving the lipid components (phospholipid and cholesterol). This solvent was removed from the media by evaporation, and subsequently, saponinosomes formed in the aqueous phase. According to the solvent injection method, saponins are expected to interact with phospholipids and cholesterol. The formation of the SRF–phospholipid–cholesterol complex is the most important point in the preparation of saponinosomes.

**Figure 2:**
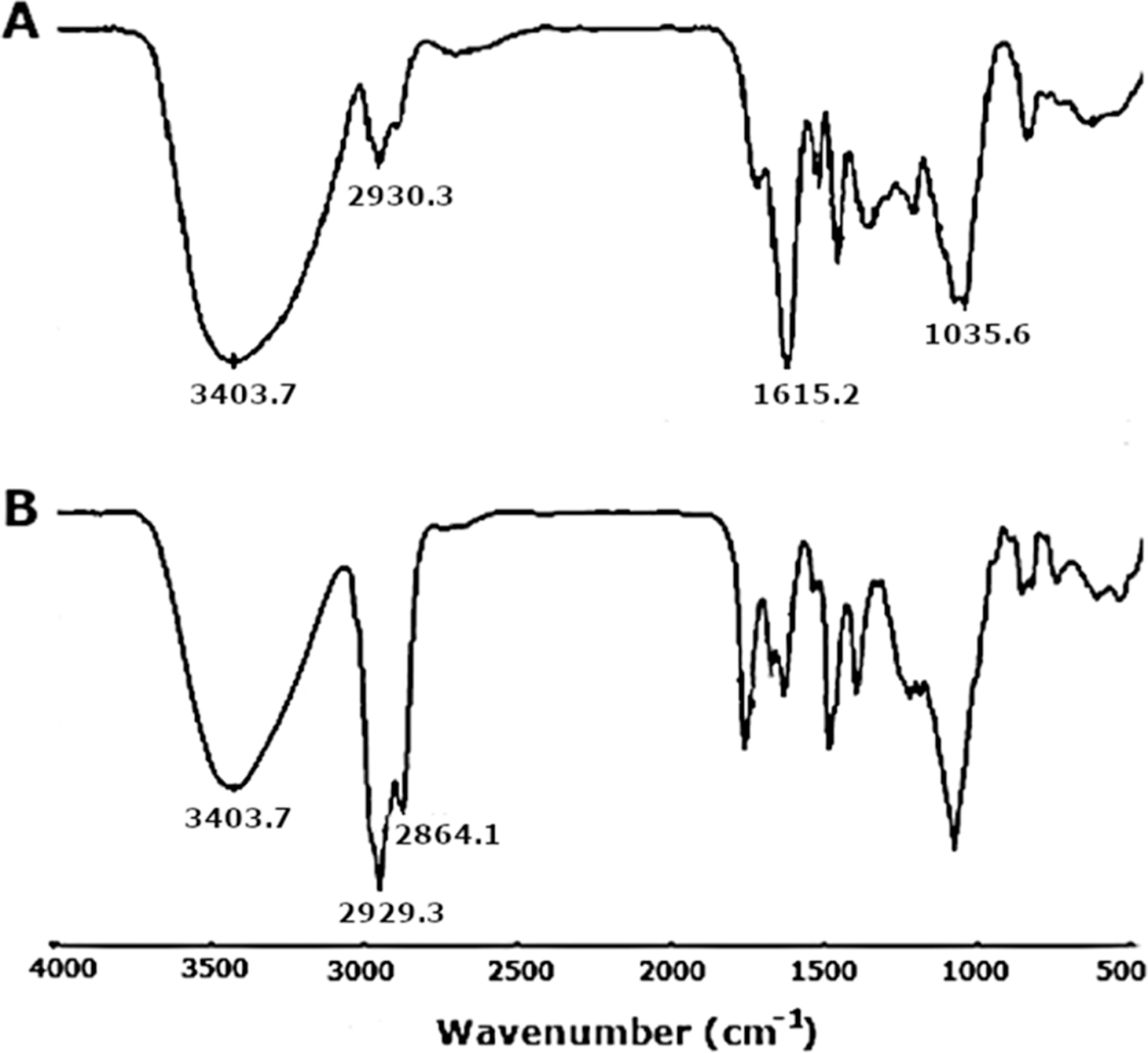
(A) FTIR spectra of the SRF and (B) saponinosomes.

The formation of the phospholipid–saponin complex was confirmed by FTIR spectroscopy (Figure 2). In addition, FTIR spectra of both phospholipid and cholesterol are given in Figure S1 (Supporting Information). The FTIR spectrum of phospholipid–saponin exhibits changes in the position of saponins and phospholipids. The saponin shows a strong peak in the region of 3404 cm^–1^ that is related to the stretching vibrations of the hydroxyl groups in the compound (Figure 2A). The peak at 2930 cm^–1^ is typical of hydrocarbons and is ascribed to the C–H stretching vibrations. The peaks in the range from 1615 to 1430 cm^–1^ are related to the C=C stretch of the unsaturated alkene and the aromatic skeletal vibrations of the extracts, respectively. The peak at 1036 cm^–1^ is attributed to oligosaccharide linkage absorption (C–O–C) in the structure of saponin.

Figure 2B shows the FTIR spectrum of saponinosomes, and the characteristic bonds of both the SRF and phospholipid are visible. The prominent peaks at 2929 and 2864 cm^–1^ in Figure 2B are related to the hydrocarbon stretching vibration of the fatty acid chains, which are a part of the phospholipid structure that is a component of the saponinosomes. The amplitudes of these peaks are much higher than those of the corresponding peaks for the bare SRF, and this is a conspicuous difference between the spectra.

A comparison of the individual peaks of the saponinosome precursors with the final complexes shows that the saponins have hydrogen bonds to the phospholipids and cholesterol in the saponinosome structure. The decrease of the OH peak amplitude in the saponinosomes, compared to that of the SRF, accounts for the formation of a hydrogen bond between the saponin and the phospholipid. It can be argued that the hydrogen bond is formed between the hydroxyl groups of the saponin with the phosphorous–oxygen group of the phospholipid. The increased vibration amplitude of the C–H bond at 2929 and 2864 cm^–1^ along with the decreased amplitude of the C=C vibration of saponin suggests that most saponin is wrapped inside the long fatty acid chains of the phospholipid. Similar results from FTIR measurements on saponins from medicinal plants have previously been reported.^52^

### Size, Zeta Potential, and Morphology

The particle size and zeta potential are important properties of nanophytosomes related to stability and reproducibility, and this information predicts the state of nanoparticles for drug delivery. The hydrodynamic diameter from DLS and the zeta potential of the unloaded formulation were found to be 50 ± 2 nm and −41 ± 5 mV, respectively, whereas for the loaded formulation, the corresponding values were 58 ± 6 nm and −32 ± 2 mV, respectively. It is natural to expect a larger size when the carrier takes up the cargo. We have no explanation for the difference in the value of the zeta potential for the unloaded and loaded formulations. However, the large negative values of the zeta potential in both cases provide the electrostatic stability to the species and prevent aggregation of the particles. The polydispersity index of the unloaded and loaded formulations was 0.11 and 0.28, respectively.

**Figure 3:**
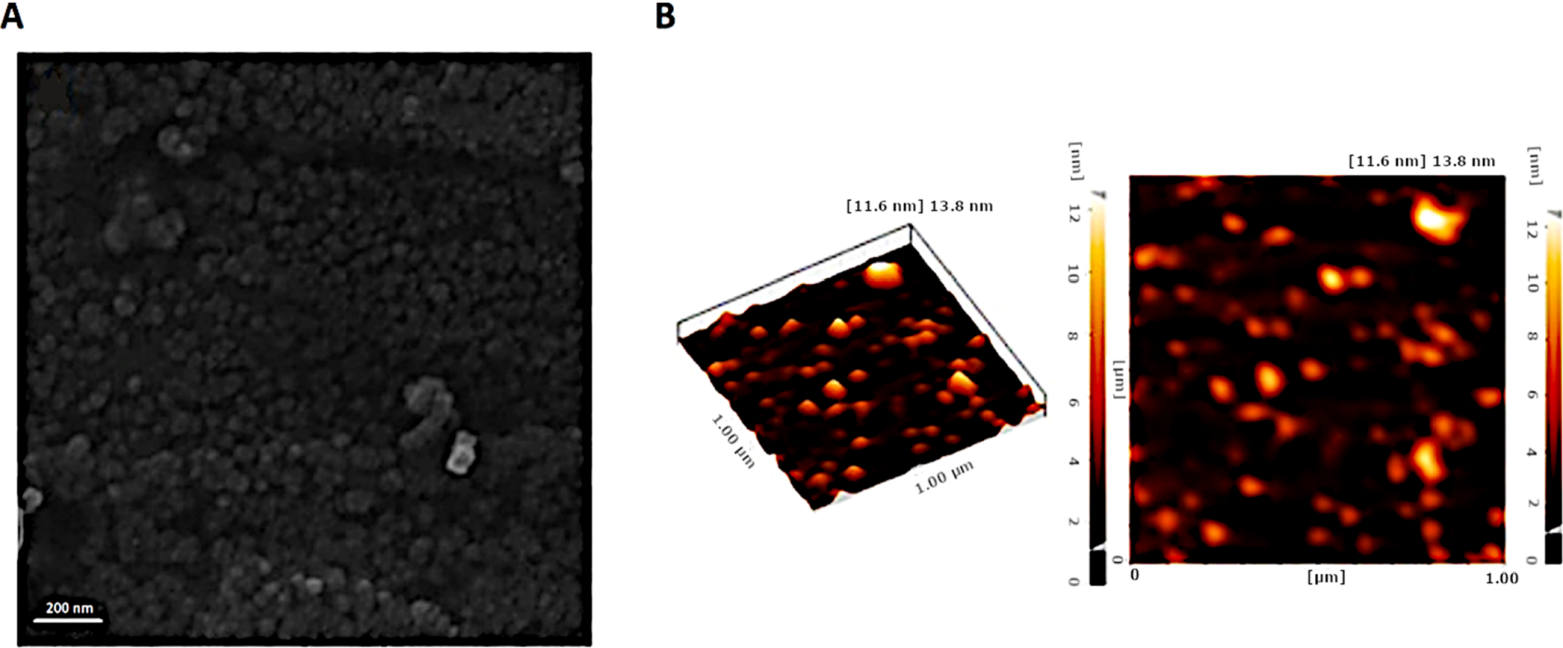
(A) FE-SEM and (B) AFM images of saponinosomes.

Morphology is another factor that may affect particle stability. SEM and AFM analyses provide information about the morphology of the prepared saponinosomes (Figure 3). The results show that the saponinosomes have a spherical shape with a smooth surface, and little agglomeration is observed. The size of the particles from microscopy is consistent with the size determined by DLS.

### Entrapment Efficiency

To estimate the amount of the loaded SRF in the saponinosomes and remove the unloaded SRF, the saponinosome samples were centrifuged at 20,000 rpm for 15 min, and the concentration of the free SRF in the supernatant was determined by measuring the absorbance at 544 nm using a UV–vis spectrophotometer. Based on our results, the EE (%) was calculated from eq 1 by using a colorimetric method. In this approach, vanillin and sulfuric acid were used as chromogenic reagents. The EE of the SRF in saponinosomes was calculated to be 85 ± 3 (%). According to this result, it seems that nanophytosomes have a relatively high EE, which makes the loading of the SRF effective. In this way, they can establish direct conjugation with lipids in their vesicle structures, allowing saponins to be easily entrapped in these saponinosomes.

### In Vitro Release Profile

One of the most important features of a drug delivery system is the ability of the nanocarrier to release the cargo at a specific site. The in vitro release of the SRF from saponinosomes was studied in PBS buffer (with a concentration of 1 M) with a pH of 7.4 using a dialysis method. The percentage of saponin released from saponinosomes at specific time intervals was calculated using the standard curve established for the SRF in PBS. The release of the SRF occurs through a combination of diffusion of the saponin from the saponinosomes to the external environment and gradual degradation of the structure. The decrease in saponin release with a longer duration indicates the importance of the diffusion process for the release kinetics.

The stability of nanocarriers in clinical applications is of great pharmaceutical importance. The most important property of phytosomes compared to other saponin carriers is their high stability. Phytosomes have a structure that resembles that of liposomes as they are both synthesized from the same compound and share some similarities. Liposomes are known to have a faster rate of degradation than phytosomes.^53^

The fundamental difference between liposomes and phytosomes is that in liposomes, the active compounds are encapsulated in the internal aqueous core or bilayer lipid. Consequently, hydrophilic drugs can be captured in the inner aqueous phase, whereas hydrophobic drugs can be encapsulated in the bilayer lipid. In contrast, in phytosomes, the phytochemicals are conjugated to the polar head of the phospholipid, become a part of the phospholipid, and form a 1:1 or 2:1 complex depending on the substance.

In liposomes, there is no hydrogen bonding between the polar group of phospholipid molecules and bioactive substances. Therefore, the plant compounds, such as saponins, encapsulate in the inner cavity of liposomes without interacting with the liposomal compounds. The phospholipid molecules surround the bioactive substances instead of making interactions through hydrogen bonds. In phytosomes, however, the phospholipid and phytoactive components form hydrogen bonds with each other at the polar parts. This action increases the stability and decreases the rate of degradation of these particles. These differences result in phytosomes having much better absorption and higher bioavailability than liposomes.^54^ It can be argued that phytosomes are generally more bioavailable than a free herbal extract due to their enhanced capacity to cross the lipid-rich biomembrane and better circulation. Phytosomes containing herbal extracts have higher absorption and bioavailability.

**Figure 4:**
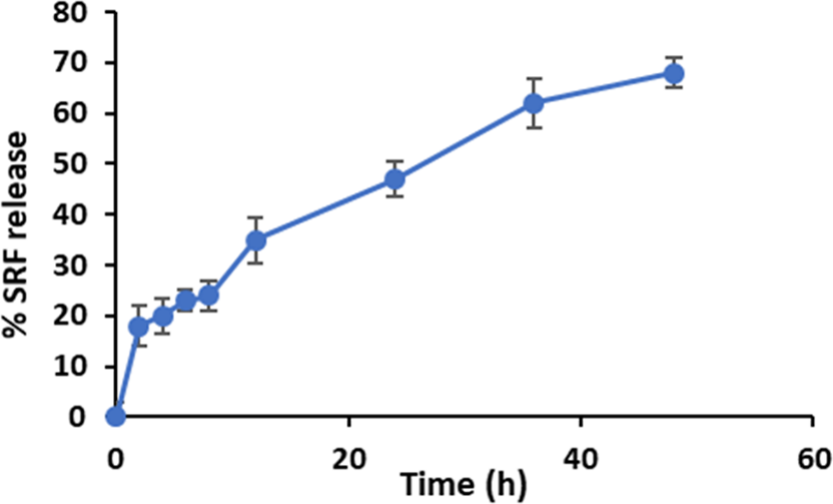
In vitro release of the SRF from saponinosomes.

Figure 4 displays the release profile of the SRF in saponinosomes under in vitro conditions. The in vitro release profile shows that 35% of the saponin was released from saponinosomes within 12 h. After the initial burst release, a moderate release was observed during the rest of the observation period. Sustained and controlled release is observed; after 48 h, 68.2% of the loaded saponin was released. The initial burst release is due to the saponin molecules attached to the surface of the saponinosomes; the sustained release is from the saponin entrapped in the carriers. The observed sustained release after the initial burst is an important feature because the controlled release is required in the field of cancer therapy. These results suggest that saponinosomes may serve as a controlled release system in cancer therapy.

### In Vitro Cytotoxicity Assay

**Figure 5:**
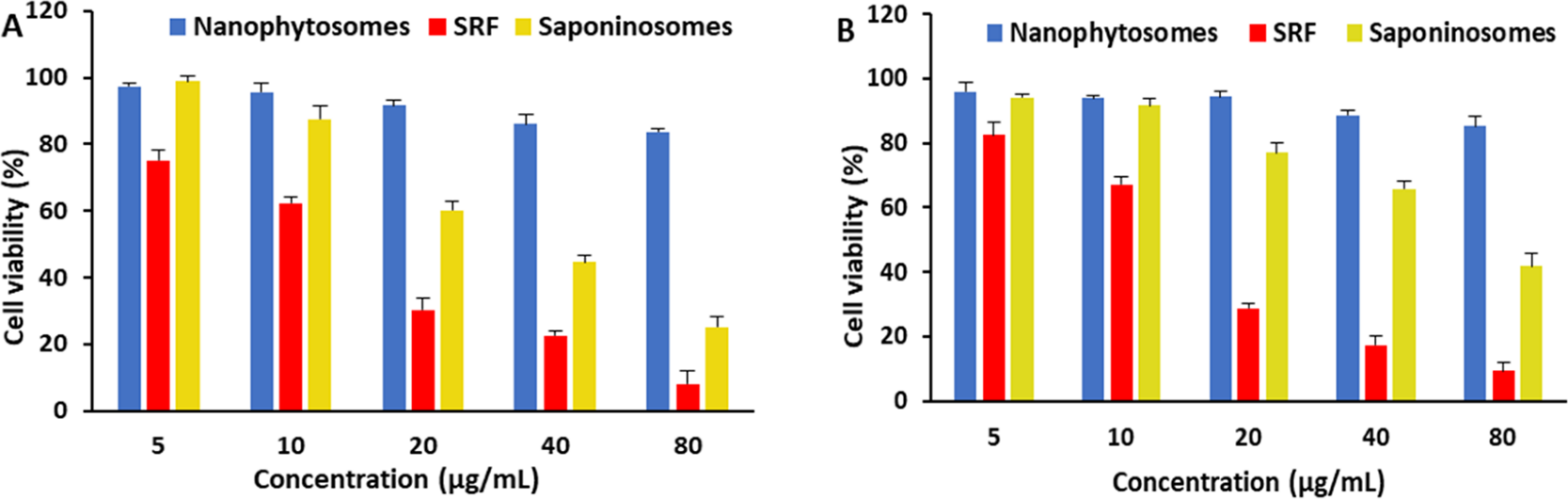
MTT assay analysis of different concentrations of the blank nanophytosomes, SRFs, and saponinosomes on the cancerous B16F10 cell line (A) and the normal L929 cell line (B).

An MTT assay was utilized to assess the in vitro cytotoxicity of the different formulations using B16F10 (mouse melanoma) and L929 (mouse fibroblast) cell lines. An important goal of this project was to reduce the membrane toxic effects of saponins on normal cells by incorporating them into stable nanophytosomes to establish saponinosome carriers. As shown in Figure 5, the viability of both cell lines in the presence of nanophytosomes or with the phospholipid complexes is larger than 80%, and the effect of the concentration is small. It can be concluded that these nanophytosomes themselves cannot inhibit the growth of tumor cells. Despite the biocompatibility of the nanophytosomes, the SRF component exhibits significant toxicity. The SRF induces considerable toxicity in both cell lines; it is clear that the cytotoxicity of the tested samples increases significantly with increasing doses. Various anticancer effects of saponins have been reported in the literature. Some studies have linked the anticancer effects to membrane permeabilization and apoptosis, but saponins have been observed to exert chemotherapeutic effects via various cytotoxic pathways.^3,18^

In the case of the saponinosome carriers, the results for both cell lines show that cell viability gradually decreases with increasing saponinosome concentration, but this trend is much stronger for the cancer cell line (B16F10) (see Figure 5). The reason for this difference may be that the blood vessels of tumors have larger pores, and therefore, vascular permeability is much higher than that in normal tissues.^55−58^ In contrast to normal vessels, tumor vessels are heterogeneous in their spatial distribution, dilated, and tortuous, leaving avascular spaces of different sizes. This phenomenon is associated with the enhanced permeability and retention effect,^59^ which is considered universal to solid tumors. This effect may enhance the penetration of nanocarriers into tumors.^58^

Saponins can interact with sterols in the cell membrane, leading to cell death (cf. the Introduction section). At corresponding concentrations, it is evident that cytotoxicity is much higher in the presence of the SRF than in the cells treated with saponinosomes. However, saponinosomes have a stronger toxic effect on cancer cells compared with that on normal cells, especially at higher concentrations (Figure 5). Saponin exposes both cancerous and normal cells to high cytotoxicity, while the saponinosome delivery system can reduce this toxic effect on normal cells and therefore may be a better option for cancer therapy.

The antitumor cell membranes and effects of saponins have received much attention in recent years.^3,7,8,55^ The important factors for the antitumor effects of saponins are pore formation with an increase in permeabilization, induction of apoptosis, inhibition of angiogenesis and metastasis, and reduction of drug efflux. The cytotoxicity of saponins to normal cells appears to be of the same order of magnitude as their effect on cancer cells through the formation of complexes with cholesterol in the cell membrane, leading to pore formation and permeabilization of cells. Since membrane toxicity is characteristic of many saponins due to their amphiphilic nature, stable incorporation into nanoparticle formulations could be a practical solution to reduce toxicity while increasing the cell-targeting potential.^60^

Some studies have shown that the SRF of *Z. spina-christi* can induce cancer cell death.^61^ However, in this study, saponinosomes were used as drug carriers to reduce the cytotoxicity of saponins to normal cells. Saponins isolated from various plants and animals have been shown to specifically inhibit the growth of cancer cells in vitro.^37^ The search for natural substances capable of combating malignancies has led to considerable research on this property of saponins. Such carriers with minimal toxicity and significant effect enhancement are ideal for safe and effective cancer chemotherapy.

### Qualitative Assessment of Cellular Uptake by Fluorescence Microscopy

**Figure 6:**
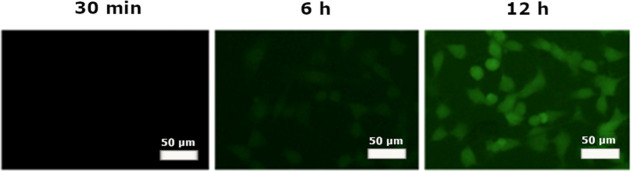
Cell internalization of F-nanophytosomes in B16F10 cancer cells after different incubation times.

To evaluate the cellular uptake of saponinosomes, fluorescence microscopy was utilized on B16F10 cancer cells treated with F-nanophytosomes (see Figure 6). No signs of cellular uptake were observed during the first minutes of incubation. However, after an incubation period of 6 h, the cancer cells showed internalization of the samples. After 12 h of incubation, an increase in fluorescence intensity was found in the fluorescence microscopy images. This indicates that the cellular uptake of nanophytosomes is a time-dependent process and that the carriers enter the cells after some time.

### Quantitative Assessment of Cellular Uptake by Flow Cytometry Analysis

**Figure 7:**
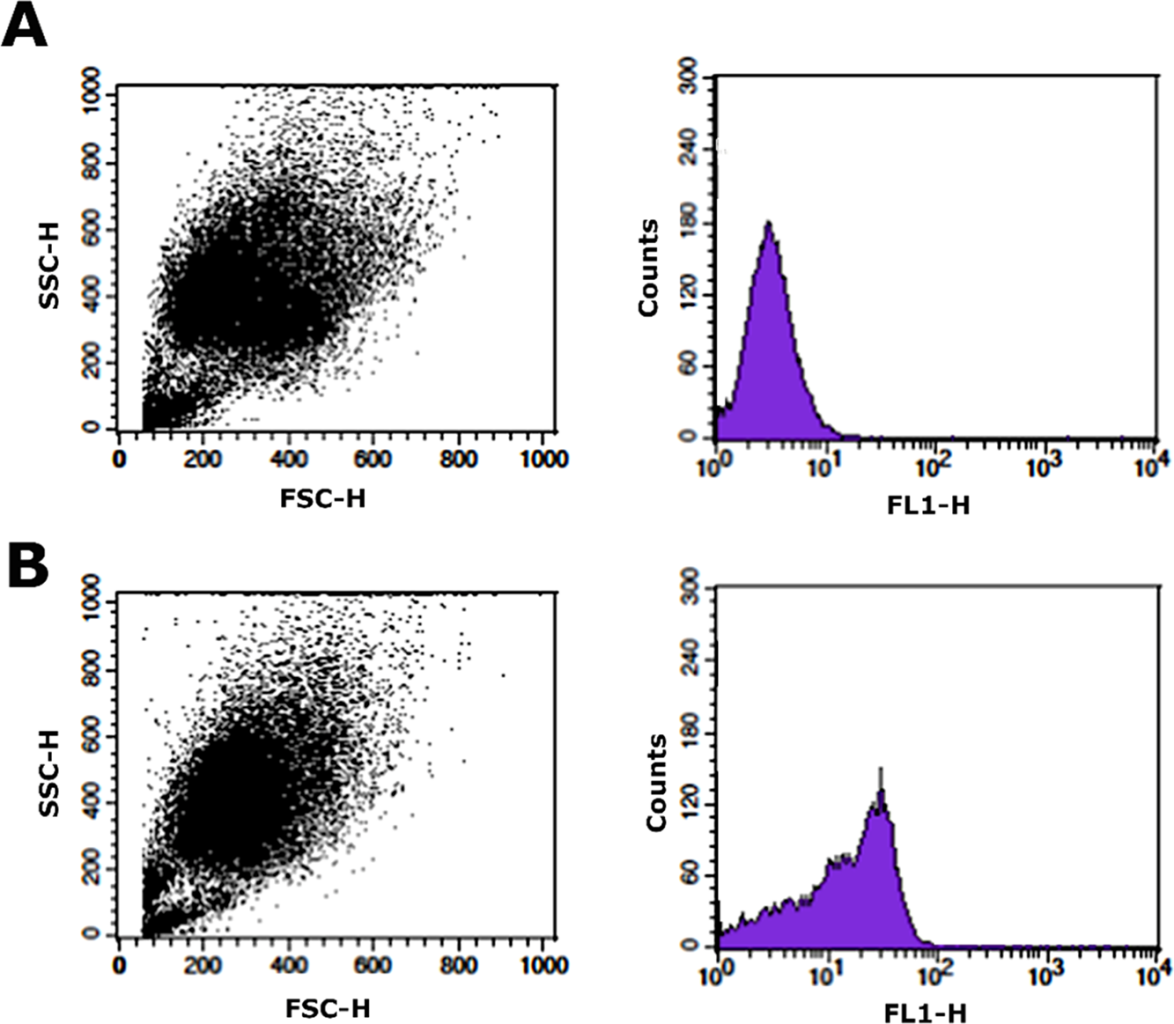
Flow cytometry analysis of B16F10 cells after 6 h of incubation for the control group (A) and F-nanophytosomes (B).

The efficacy of the internalization of saponinosomes by B16F10 cells was determined by flow cytometric analysis (see Figure 7). Figure 7A shows the auto-fluorescence of the B16F10 cells dispersed in PBS buffer (control group), and the fluorescence intensity is shown in the histogram on the right-hand side. The significant rightward shift in the histogram in Figure 7B indicates that the F-nanophytosome carriers are efficiently taken up by the cancer cells.

### Evaluation of the Efficacy of Saponinosome Therapy in Inhibiting Melanoma Tumor Growth

**Figure 8:**
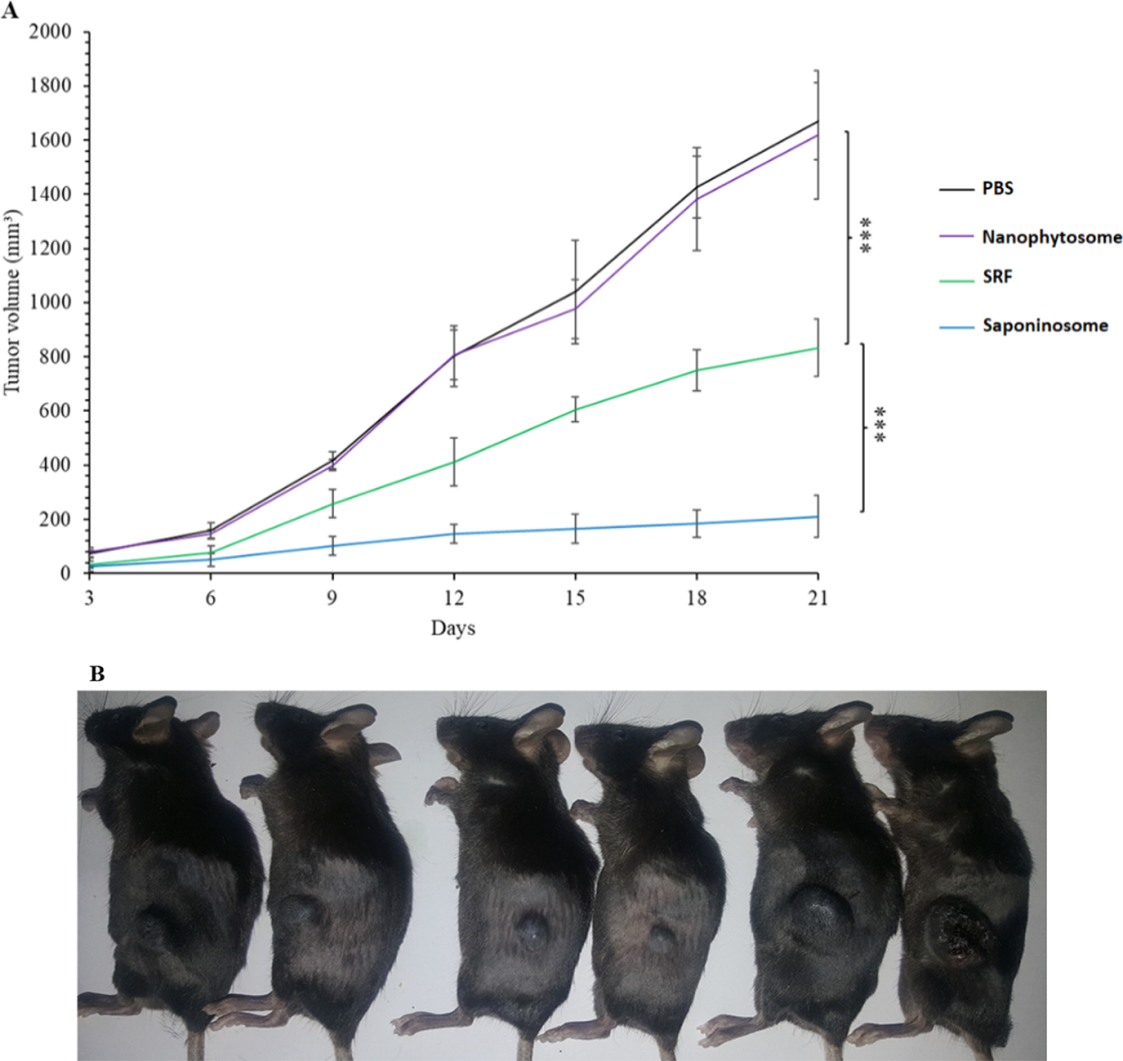
Evaluation of treatment of mice bearing B16F10 melanoma tumor with different formulations. (A) Evolution of the tumor volume in the different treatment groups. (*: *P* ≤ 0.05, **: *P* ≤ 0.005, ***: *P* ≤ 0.001, ns: not significant.) (B) Recorded image of treated mice in different groups (from right to left: first, PBS; second, blank nanophytosomes; third and fourth, saponinosomes; and fifth and sixth, SRF).

B16F10 melanoma cancer cells were subcutaneously injected into C57BL/6 mice, and tumor growth was evaluated in different treatment groups (see Figure 8). The different systems of PBS, SRFs, nanophytosomes, and saponinosomes were injected intraperitoneally into the mice every other day for 21 days. The development of the tumor was monitored every third day. Figure 8A shows the development of tumors over a 21 day period, and the tumor volume is measured using eq 3. In the presence of PBS and blank nanophytosomes, tumors grow rapidly, and the growth is virtually unaffected. However, when the SRF is added, growth is significantly inhibited, demonstrating that *Z. spina-christi* saponin has considerable antitumor properties in the treatment of melanoma in mice. Interestingly, compared to all other systems, saponinosomes show the most efficient anticancer effect, even better than that of the SRF. In this case, tumor growth is almost completely suppressed. This suggests that saponinosomes exhibit better tumor penetration and tumor accumulation than the small free drug molecules. We assume that the small drug molecules are widely distributed in the bloodstream, whereas the saponinosomes are stronger localized in the tumors. In addition, it is possible that the hydrophobicity of the saponins is higher than that of the saponinosomes, and therefore, the circulation time of the saponins in the blood is shorter before they are taken up by macrophages. In a recent study^62^ with mice bearing an H22 tumor, it was shown that doxorubicin-loaded exosome-biomimetic nanoparticles reduced the tumor volume much more efficiently than free doxorubicin.

### Evaluation of Biocompatibility Aspects

**Figure 9:**
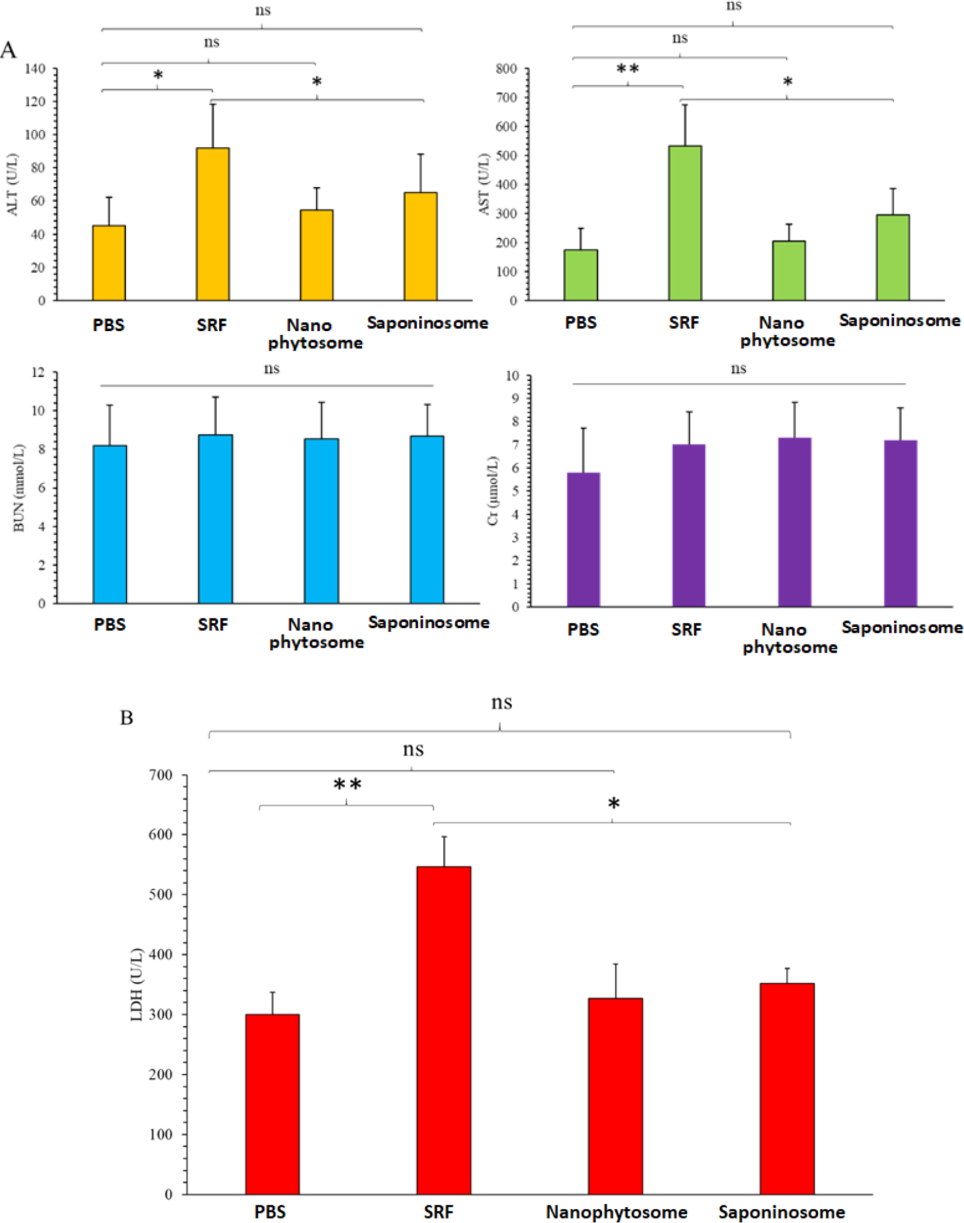
Biochemical blood test to evaluate the biocompatibility of the different formulations. (A) BUN, Cr, AST, and ALT in the different groups of treated mice and (B) LDH levels of plasma to evaluate the hemolysis range.

Biocompatibility is one of the most important aspects of drug delivery systems. Therefore, this property was investigated in more detail in this study. C57BL/6 mice were treated with an intraperitoneal injection of the different formulations every other day for 21 days. Then, after 21 days of treatment, they were sacrificed, and their blood was collected by cardiac puncture to study the blood biochemical factors such as BUN, Cr, AST, ALT, and LDH. The results are shown in Figure 9. The significantly higher values of AST and ALT for the SRF than for the other systems indicate that liver injury is more pronounced with the SRF than with the other systems, and high values of AST and ALT indicate hepatocyte injury.

To obtain information about hemolysis,^62^ LDH was measured in the blood plasma of mice (Figure 9B). It is obvious that the LDH level is much higher for the SRF than for the other systems. In hemolysis, a high LDH value indicates that many cells in the intravascular space have been destroyed. The values of BUN and Cr are virtually the same for all systems tested, indicating that the kidneys are not more damaged by any of the systems.

### Histopathological Examination

**Figure 10:**
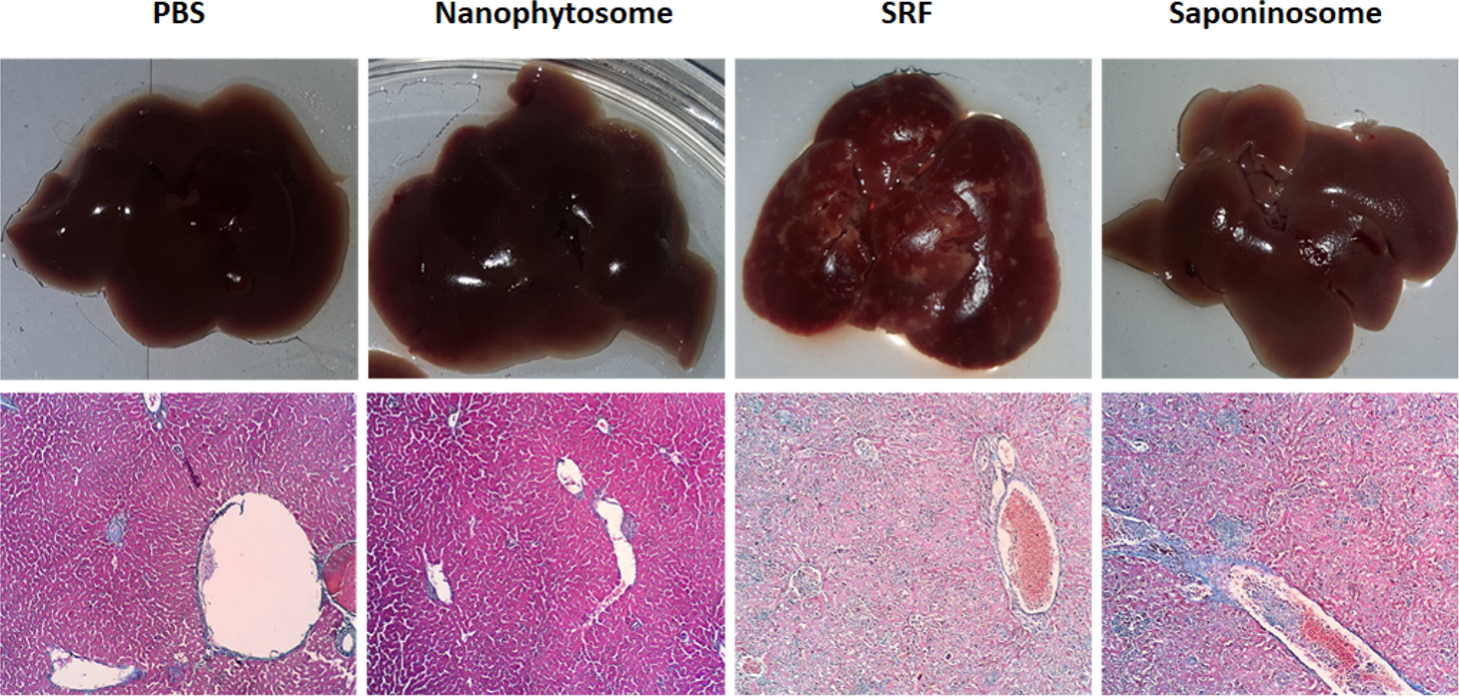
Histopathological examination of the mouse liver to assess the extent of damage.

One of the known side effects of saponin is hepatotoxicity. Figure 10 shows the effects of the different treatments on the liver of mice. The results of the histopathological test show that saponin causes the most severe impairment of hepatocytes and eventually fibrosis. This side effect is less prominent when the liver is treated with saponinosomes. After injection of the SRF, several fibrotic areas were observed on the liver surface; this side effect was not present in mice treated with the other formulations. Thus, the saponinosome system not only increases the therapeutic and antitumor effects of saponin but also improves its biocompatibility and reduces its side effects such as hepatotoxicity.

## Conclusions

The constituents of plants, especially saponins, are gaining increasing attention because of their antitumor effects. However, the cytotoxicity of saponins is an obstacle to their use as anticancer agents in clinical trials. In this work, an SRF was prepared from the leaves of *Z. spina-christi*. The carrier for the SRF is phytosomes; we refer to the SRF-loaded phytosome as a saponinosome, and this is the first time that this concept is used, and this carrier has not been used before for anticancer therapy. The size of a saponinosome is about 60 nm and the zeta potential is ca. −30 mV, and the FE-SEM and AFM measurements show that the saponinosomes are spherical in shape and the surface is smooth.

The release profile of the saponin in vitro indicates that the saponin is released in a controlled manner, and approximately 68% of the cargo was released within 48 h. The cytotoxicity experiments on B16F10 and L929 cell lines show that both saponin and saponinosomes reduce the viability of both cancer cells and normal cells, but the toxicity to normal cells is much lower in the presence of saponinosomes, even at higher concentrations. This is a great advantage for cancer therapy.

Using fluorescence microscopy, the cellular uptake of fluorescein-labeled saponinosomes was observed after approximately 6 h of incubation; this time-dependent process continued for 12 h. In addition, flow cytometric analysis of B16F10 cells was performed. These experiments confirmed that the carriers were efficiently taken up by cancer cells, and in vivo studies in mice with tumors revealed that tumor volume growth was inhibited over time by the injection of saponin or saponinosomes. However, the effect was significantly greater with saponinosomes; tumor growth was almost completely suppressed in this case. This demonstrates that saponinosomes are more efficient than the free drug (saponin) in cancer treatment.

To evaluate the biocompatibility of the different treatment systems, the biochemical factors in the blood were studied. The much higher levels of AST, ALT, and LDH in saponin treatment indicate that liver damage and hemolysis are more pronounced when the body is exposed to saponin. The levels of BUN and Cr are the same for all systems, suggesting that the kidneys are not particularly damaged by any of the systems. Looking at the liver from a histopathological point of view, the results show that the saponin damages hepatocytes the most and eventually leads to fibrosis. The results of this work clearly show that the saponin has a promising potential as an anticancer drug and that saponinosomes act as efficient carriers of this drug.

## Supporting Information Available

FTIR results for the SRF, phospholipid, cholesterol, SRF–phospholipid complex, SRF–cholesterol complex, and saponinosome (PDF)
