# Supplementary material for: Use of Saponinosomes from Ziziphus spina-christi as Anticancer Drug Carriers
Source: ACS Omega. 2022 Aug 4;7(32):28421–33. doi: 10.1021/acsomega.2c03109 (PMC9386697; doi:10.1021/acsomega.2c03109)
Supplement: Supplementary file 1 — ao2c03109_si_001.pdf [file ao2c03109_si_001.pdf]

Supporting information

Use of Saponinosomes from Ziziphus Spina-Christi as Anti-Cancer Drug Carriers

Zahra Nazemoroaya, Mohsen Sarafbidabad*, Athar Mahdieh, Darya Zeini, and Bo Nyström*

Zahra Nazemoroaya
Student Research Committee, School of Pharmacy, Shahid Beheshti University of Medical Sciences, Tehran, Iran
E-mail: z.nazem@sbmu.ac.ir

Mohsen Sarafbidabad
Department of Biomedical Engineering, Faculty of Engineering, University of Isfahan, Isfahan, Iran
E-mail: m.saraf@eng.ui.ac.ir

Athar Mahdieh
School of Pharmacy, Department of Pharmaceutics, University of Oslo, P.O. Box 1068, Blindern, N-0316 Oslo, Norway; Department of Chemistry, University of Oslo, P.O. Box 1033, Blindern, N-0315 Oslo, Norway
E-mail: athar.mahdieh@farmasi.uio.no

Darya Zeini
Department of Chemistry, University of Oslo, P.O. Box 1033, Blindern, N-0315 Oslo, Norway
Laboratory of Neural Development and Optical Recording (NDEVOR), Department of Molecular Medicine, Institute of Basic Medical Sciences, University of Oslo, P.O. Box 1103, Oslo, Norway

E-mail: darya.zeini@medisin.uio.no

Bo Nyström
Department of Chemistry, University of Oslo, P.O. Box 1033, Blindern, N-0315 Oslo, Norway
E-mail: b.o.g.nystrom@kjemi.uio.no

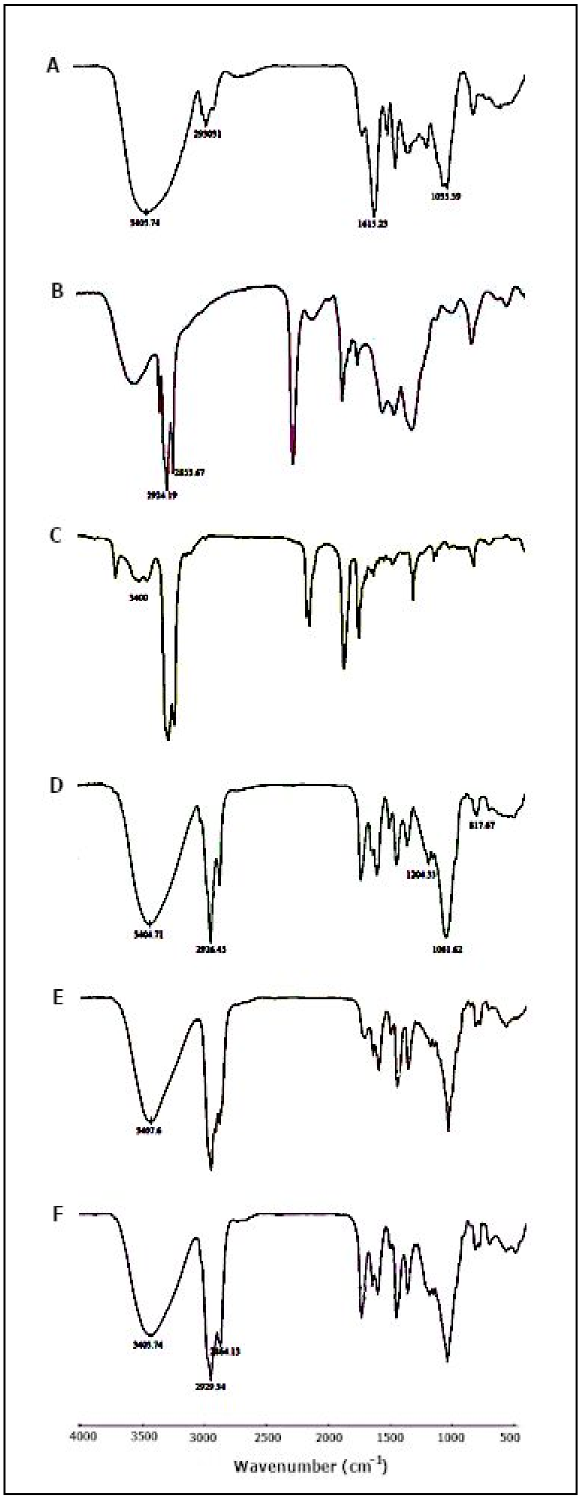

Figure S1. FTIR spectra of SRF (A), phospholipid (B), cholesterol (C), SRF-Phospholipid complex (D), SRF-Cholesterol complex (E), and Saponinosome (F).
